# “We talk it over” - mixed-method study of interdisciplinary collaborations in private practice among urologists and oncologists in Germany

**DOI:** 10.1186/1471-2407-14-746

**Published:** 2014-10-04

**Authors:** Sandra Beermann, Denny Chakkalakal, Rebecca Muckelbauer, Lothar Weißbach, Christine Holmberg

**Affiliations:** Foundation of Men’s Health, Berlin, Germany Claire Waldoff-Straße 3, 10117 Berlin, Germany; Berlin School of Public Health, Charité - Universitätsmedizin Berlin, Seestr 73, Haus 10, 10117 Berlin, Germany

**Keywords:** Urology, Oncology, Interdisciplinary collaboration, Community setting, Multidisciplinary

## Abstract

**Background:**

Utilisation of multidisciplinary teams is considered the best approach to care and treatment for cancer patients. However, the multidisciplinary approach has mainly focused on inpatient care rather than routine outpatient care. The situation in private practice care and outpatient care is gradually changing. We aimed to 1), investigate interdisciplinary cooperations in the care of tumor patients among urologists and oncologists in the community setting, 2), establish an estimate of the prevalence of cooperation among oncologists and organ-specific providers in community settings in Germany and 3), characterise existing cooperations among oncologists and urologists.

**Methods:**

We conducted simultaneously a cross-sectional survey with private practice urologists (n = 1,925) and a qualitative study consisting of semi-structured interviews with urologists and oncologists (n = 42), primarily with private practices, who had indicated cooperation the care of urological tumor patients.

**Results:**

Most of the participants (66%) treated their own tumor patients. When physicians referred patients, they did so for co- and subsequent treatments (43%). Most cooperating urologists were satisfied with the partnership and appreciated the competency of their partners. Qualitative interviews revealed two types of collaboration in the community setting: formal and informal. Collaborations were usually ongoing with many physicians and depended equally on both patient preference and diagnosis.

**Conclusion:**

Joint patient treatment requires clear delineation of roles and responsibilities and simple means of communication. Formal frameworks should allow for incorporation of patients’ critical role in collaboration decisions in treatment and care.

## Background

Utilisation of multidisciplinary or multi-professional teams in cancer care and treatment is presently considered the best approach for cancer patient care [1–5]. Comprehensive reforms were necessary to facilitate multidisciplinary care and have been completed or are under way in the organisational structure of health care delivery for oncology patients around the world [4, 6–9]. These revolutionary changes in health care delivery transformed health care systems based formerly on individual physicians’ decision-making into institutionally supported team-based approaches to treatment and care [10–12]. Multidisciplinary cancer care has improved patients’ disease management and treatment [13, 14]. However, the shift towards multidisciplinary approaches to cancer care has largely focused on inpatient care rather than on routine outpatient care. Similarly, research on multidisciplinary approaches has primarily focused on hospital settings as well as on communication within teams, particularly in team meetings, in order to investigate the influence on treatment decision-making [10, 12, 15–17]. This may be a function of how care is delivered to oncology patients in different countries. In Germany oncological care including systemic therapy is increasingly taking place via outpatient care delivered by private practices rather than within hospitals. Historically, parallel structures of specialized care in patient health care delivery existed. This was divided into hospital care and community-based private practice care consisting of general practices and specialized practices. Most patients in Germany are covered by statutory health insurance that reimburses oncological treatments both in hospital settings and private practice.

Such a strict divide in hospital and private practice care is no longer feasible due to changes in treatment and care of cancer patients. To enable and ensure interdisciplinary care in the community setting organ-specific tumor-centres today bridge the gap between private-practice and hospital-based care by incorporating both into their structure. However, while most hospitals today deliver cancer treatment through inter- and multiddisciplinary teams including psychologists, radiologists, oncologists, and organ-specific specialties, it is unknown how cancer care is delivered once patients have left the hospital [18]. In Germany oncologists and radiologists are two separate medical sub-specialties. Historically, care in private practice is single-physician based, thus a shift towards team-based approaches may be more difficult to implement in such an environment and the necessary adaption of private practice care is only gradually moving forward [19, 20]. To encourage collaborative efforts both within private practices and between private practices and hospital-based care, the legal frame of how health insurance companies reimburse private practices for their cancer care has been restructured to include incentives for collaborations [21–23]. Incentives were mostly based on higher reimbursement rates for physicians who maintain a certain level of conducting systematic therapies in their practices. These incentives were developed albeit little knowledge of actual private practice care of cancer patients at the time, and such a narrow approach has led to tensions between urologists and oncologists [21, 24–28]. How interdisciplinary collaboration among physicians is organized outside hospital settings is still in question. To investigate cooperations in the care of tumor patients in community settings we aimed to establish an estimate of the prevalence of cooperation among private practice oncologists and urologists and characterize how they collaborate in community settings in Germany. We particularly focused on the cooperation of urologists because the shift towards multidisciplinary work for urological tumors is fairly recent [29, 30].

However, in contrast to multidisciplinary team approaches to cancer care in other countries, in Germany current efforts to foster collaborations among private practices caring for oncological patients focus on physicians of various relevant disciplines rather than on the inclusions of other types of health care providers. In general, patients in Germany are free to choose which practices deliver their care.

## Methods

We simultaneously conducted a cross-sectional survey with private practice urologists to estimate the prevalence of cooperation and a qualitative study consisting of semi-structured interviews with private practice urologists and oncologists who had indicated that they cooperated with each other in urological care to characterize cooperations in the community setting. The study was approved by the Charité-Universitätsmedizin Berlin ethics committee (EA2/165/11).

### Quantitative study component

#### Study sample

For the cross-sectional survey we included urologists who worked in private practice in Germany in 2011; there are approximately 7,000 licensed urologists [3]. Out of these 3,500 were members of the Federal Association of German Urologists (BDU) in 2011. More than 60% of the BDU members (n = 1,925) operated from a private practice as of March 2011.

#### Data collection

We invited these 1,925 urologists via mail from the Foundation for Men’s Health, a German nongovernmental organisation, that included a letter introducing the study, questionnaire and postage paid envelope. Also included was an ID-coded postcard to be sent separately to track respondents and ensure anonymity. In accordance with the Dillmann method, those who had not sent back the postcard after four weeks were sent a reminder postcard [31]. A replacement questionnaire was sent out an additional four weeks later to remaining non-responders to increase participation rates. Those who had not responded four weeks after the replacement questionnaire had been sent out were considered non-responders.

The questionnaire was developed based on the aims of the study and a literature review on factors that influence and structure cooperations among physicians. It was tested and improved via cognitive interviewing and then pre-tested with urologists who owned a private practice.

The final survey consisted of 31 questions that were divided into three sections: 1), sociodemographic information of the physician and their private practice clientele, including patient volume at the office, number of patients with urological tumors, and extent of chemotherapy administered in the office, 2), urologist’s referral behaviour, to whom patients were referred and why, 3), prevalence and characteristics of existing cooperations with oncologists.

#### Statistical analysis

For the descriptive data analysis, we used mean and standard deviation (SD) for continuous variables and percentages for categorical variables. To investigate which factors were associated with the prevalence of an existing cooperation we used multivariable logistic regression. As independent variables we considered age of the urologist (in years), gender (male/female), the region in which the urologist practices (West Germany/East Germany), and the size of the municipality where the urologist’s practice is located (divided into three categories by size: small towns with <20,000 inhabitants, mid-size towns with 20,000-100,000 inhabitants, or larger towns with >100,000 inhabitants), and whether they were sole operators of their practice or worked in a joint practice and whether they were members of an interdisciplinary tumor centre (yes/no). Correlation analyses showed that no colinearity was prevalent between the independent variables, an assumption for the logistic regression.

All statistical analyses were performed using Stata IC version 12. P values <0.05 were considered statistically significant.

### Qualitative study component

#### Study sample

For the qualitative study, we selected tandems of cooperating urologists and oncologists. To identify such tandems, 49 members of the Scientific Institute of Private Practice Hematologists and Oncologists were contacted and screened for participation. In addition, the Foundation for Men’s Health contacted urologists to screen them for participation; these physicians were asked to indicate with whom they collaborated in the care of patients with urological tumors. If an oncologist and an urologist indicated each other they were invited to participate in the interview study. In addition, to reflect the practice of urological care in Germany in which much of the care takes place between generalising urologists and urologists specialised in oncology care, a select sample of urologists who specialised in oncology care and performed intravenous chemotherapies were asked to participate in the interview study. To reflect cooperations between hospitals and private practice urologists we invited two chiefs of medicine from oncology hospital departments to join the interview study.

#### Data collection

Twenty-one identified pairs of collaborating urologists and oncologists were interviewed individually. The interview guideline was developed based on the study aims and the literature review. It included questions regarding the practice, cooperation with the other physician and operational framework for cooperation including related conditions, barriers and enabling factors of the particular cooperation and of cooperations in general. To ensure anonymity of participants, interviewees were assigned identification numbers with which transcribed interviews were pseudonymised. All interviewees signed informed consent forms.

#### Analysis

Interview transcripts were entered into MAXQDA 10 and analyzed thematically with regards to tandem cooperation specifically between the two physicians, any other collaborations they discussed, and barriers and/or enabling factors for collaborations with other physicians. Codes were developed inductively from the interview material. After an initial round of coding, categories were developed and codes were grouped within the categories. Two authors (CH and DC) coded materials and resolved differential coding. In addition to thematic coding, the interviews of the tandems were compared to each other; a case report developed for every tandem was discussed by the two coders. All steps of analysis were regularly presented and discussed within a qualitative working group at the Charité-Universitätsmedizin Berlin.

## Results

### Quantitative study component

#### Sample characteristics

The overall questionnaire response rate was 40% (n = 731). Of the responding urologists, 92% were male. The average age of participants was 51.8 years (SD: 6.8). Most of the physicians worked as sole operators (48%) and 44% worked in a joint practice. On average, they had operated their practice for 14.4 years (SD: 7.1). Most of the participants practiced in towns with 20,000-100,000 inhabitants (38%) or in towns with more than 100,000 inhabitants (38%) whereas 22% practiced in more rural areas (i.e., in towns with less than 20,000 inhabitants). 63% of responders were members of an interdisciplinary tumor centre. During the fourth quarter of 2011, 76% of participants had diagnosed at least one urological tumor; 50% of participants had administered between one and ten chemotherapies while the remaining 50% of the sample had not performed any chemotherapy.

#### Referrals

Most participants (66%, n = 482) treated their tumor patients themselves. Of the remaining responders (n = 249), 15% referred their patients to a specialised urologist, 9% to an oncologist, and 10% to a clinic. The reasons for transferring patients were treatment-related (43%), to seek a second opinion (15%) or for a consultative examination (3%).

#### Collaborations

Reasons in favour of or opposition to cooperations with oncologists or with oncology-qualified urologists are displayed in Table 1.

**Table 1:** **Urologists’ reasons for or against cooperation with oncologists or urologists specialised in oncology care** ^**a**^

Reasons	All participants (n = 731) n (%)
**In favour of cooperation**	
To offer additional treatments	242 (31.1)
To improve patient care	264 (36.1)
It is mandated by the health insurance company	77 (10.5)
**Opposed to cooperation**	
I can offer all treatments needed by patients with urological tumors	291 (39.8)
I have had negative experiences with collaborations	14 (1.9)
I am worried I would lose patients	2 (0.3)

^a^Numbers do not sum up to 100% because multiple answers were allowed.

A total of 437 physicians (59.8%) stated that they collaborated with oncologists or other urologists (Table 2). Most of the cooperation partners knew each other. Together they decided on treatment and supervised the patient. Follow-up care remained with the urologist in the majority of cases (Table 2). Most of the cooperating urologists were satisfied (40%) or very satisfied (45%) with the partnership. They especially appreciated the competency of their partners (81%). The urologists appreciated the non-bureaucratic exchange with their colleagues (72%) and the clear distribution of roles and responsibilities for care (35%).

**Table 2:** **Description of existing cooperation**

Characteristic of the cooperation	Participants with existing cooperation (n = 437)
	***n***(%)
**Most frequent cooperating partner**	
Oncologist	155 (35.5)
Urologist specialised in oncology	260 (59.5)
No information	22 (5.0)
**Acquaintance with cooperation partner**	
I am well acquainted with my cooperation partner	343 (78.5)
I am slightly acquainted with my cooperation partner	62 (14.2)
I don’t know my cooperation partner in person	8 (1.8)
No information	24 (5.5)
**Distance to cooperating partner**	
In same building	29 (6.6)
In walking distance (≤3 km)	98 (22.4)
Near distance (3–10 km)	153 (35.0)
Moderate distance (11–30 km)	108 (24.7)
Far distance (>30 km)	25 (5.7)
No information	24 (5.5)
**Who determines the treatment?**	
I determine the treatment	64 (14.6)
My colleague determines the treatment	16 (3.7)
My colleague and I determine together	338 (77.3)
No information	19 (4.3)
**Who administers the therapy?**	
I supervise/administer the therapy	52 (11.9)
My cooperation partner supervises/administers the therapy	99 (22.7)
My cooperation partner and I jointly supervise the therapy	264 (60.4)
No information	22 (5.0)
**Who is responsible for follow-up care?**	
I am responsible for follow-up care	270 (61.8)
My cooperation partner is responsible for follow-up care	11 (2.5)
My cooperation partner and I are both responsible	134 (30.7)
No information	22 (5.0)

Logistic regression analyses clarified factors associated with likeliness of establishing cooperation (Table 3). Urologists in West Germany were less likely to cooperate (OR = 0.51, p = 0.003) as compared to urologists in East Germany. Urologists working as sole operators of their practice were more likely to cooperate (OR = 1.54; p = 0.016). Membership to a tumor centre increased the likelihood to cooperate (OR = 1.85; p < 0.001). We did not find a significant association between the prevalence of an existing cooperation and the urologists’ age and gender, the size of the town, or the number of oncological treatments in a practice.

**Table 3:** **Factors associated with the likelihood of establishing a cooperation** ^**a**^

Variables	Characteristic	OR (95% CI)	P value
Age (years)		1.02 (1.00; 1,05)	0.056
Gender	Male	0.81 (0.42; 1.59)	0.546
	Female	[Reference]	
Region	West Germany	0.51 (0.33; 0.79)	0.003
	East Germany	[Reference]	
Sole operator of private practice	Yes	1.54 (1.08; 2.19)	0.016
	No	[Reference]	
Size of the town	>100,000 inhabitants	1.50 (0.96; 2.35)	0.074
	20,000-100,000 inhabitants	1.11 (0.73; 1.69)	0.620
	<20,000 inhabitants	[Reference]	
Member of a tumor centre	Yes	1.85 (1.32; 2.60)	0.000
	No	[Reference]	
Number of treated patients	1001-1500 patients	0.99 (0.65; 1.50)	0.962
	> 1500 patients	0.80 (0.50; 1.29)	0.367
	≤1000 patients	[Reference]	

^a^Number of included participants: 626.

CI = confidence interval, OR = odds ratio.

### Qualitative study component

Of 49 oncologists, 25 agreed to participate in the qualitative study section. Of those a sub-sample of 12 could be matched with a cooperating urologist. Of 20 additionally contacted urologists, 15 agreed to participate and a matching team of 6 urologists and oncologists could be established. This led to 18 urologist-oncologist tandems for the study. Of 10 urologists who specialise in intravenous chemotherapy, 3 tandems could be identified. Thus an overall sample of 21 matched sets of urologists and oncologists participated in the study.

The tandem partners were not necessarily in close nor exclusive collaboration. All interviewed physicians were collaborating with many physicians. With whom they collaborated at any time depended on the disease, the type of treatment, and patient preference.

### Collaboration: formal and informal

Physicians described two types of collaborations. *Informal collaborations* included patient referral for additional treatments or diagnostic work-up to offices specialized in cancer care and open to discussing patient treatment options. *Formal collaborations* were usually characterised by contracts between one or several private practices and hospitals, with the exception of one case which only involved private practices.

#### Informal collaborations

For informal collaborations the urologist functioned as gatekeeper for the patient, choosing when and with whom to collaborate and when to offer a referral to the patient. Urologists decided whether or not to include a second physician based on: 1), diagnosis the patient was given, 2), stage of the tumor, and 3), type of therapy prescribed.

This involved flexible cooperation partners; physicians to which a patient was referred varied case by case. Choosing whom to collaborate with depended on preferences of both physician and patient.

*“This is also decided by the patient. It depends on accessibility and the patient’s place of residence. (…) The patient is offered different options when we discuss outpatient administration of chemotherapy. The patient then participates in the decision.” (Urologist 12B).*

*“Proximity [of treatment] is always good but it is not absolutely necessary. If this isn’t the case, it’s also okay. But you need to offer the patient the options: ‘Do you want to go someplace near where you live if it is feasible?’ Everyone has to decide that on their own.” (Urologist 9B).*

Urologists collaborated most often with oncologists for systemic therapies, particularly intravenous chemotherapy which required several employees to handle the delivery of treatment. Physical constraints on personnel led urologists to refer patients to oncologists for complex systemic treatments.

*“So for example the manpower I have. If I have a young man with testicular cancer, tumor Stage 2C, bulky disease, who needs combination chemotherapy, four cycles. You really need to be accurate there. You have to make sure the timing of the cycles is okay. You cannot allow times when this is not possible [to administer the chemotherapy]. For me in my private practice to do that, there are clear limitations. I once had three testicular cancers at one time; you need to plan for all of them. You can’t just say, ‘sorry, I don’t have time, we will only do three cycles’. Or, ‘I am on my vacation; we will just do the cycle two weeks later.’ You can’t do that. And that is why you really need to be part of a network.” (Urologist 8B).*

#### Formal collaborations

At the time of the interviews formal collaborations were only in a developmental stage, mainly consisting of participation in tumor boards organised by hospitals and private practices from different areas. In some cases the oncologist had formally agreed to administer therapies for the tandem urologist’s clientele. These arrangements were in part a response to recent changes in the reimbursement scheme for private practice physicians that treat cancer patients.

While tumor boards were the focal point of formal collaborations, varying views about how tumor boards should be organised, including how and which patients should be discussed, was a point of discussion in the interviews. Physicians discussed the issue of time and case selection for the tumor boards. The presentation of all tumor patients required a significant time commitment of physicians, so that this was not necessarily seen as the best approach. However, some oncologists feared the selection of cases presented at the tumor board might be presented too late to the other specialists.

*“There are some clearly defined situations when the tumor stage tells you what to do. Those should not be discussed at length in tumor boards.” (Oncologist 6A.)*

*“It does not make sense to discuss standard cases. (…) The cases in which you can really discuss different alternatives, additional diagnostic tests- those are really interesting. These are the rare cases. That makes tumor boards interesting.” (Urologist 11B).*

*“Maybe it would be good if tumor boards would be held more often. That would be a possibility. However, we already have plenty of them, and it may just be too much. (…) And sometimes it would be good if we were asked before a treatment regimen is decided, before they realise the treatment is not working as intended. And only then is one asked to consult on the case. It would be nice if one were asked before and decided on a treatment together. So if we would discuss patients before they start treatment. But reality often looks different than that.” (Oncologist 15A).*

*“When patients have been treated for years by the urologist even though it was clear that the course of the disease will be deadly and only when they reach the final stage then they come to us without being able to build up a relationship in this final phase. (…). So I would appreciate that if it becomes obvious that hormonal therapy is not working patients are transferred quickly so that we can build a relationship and organise treatment.” (Oncologist 11A).*

### Characteristics of collaborations that were perceived favourably

Physicians emphasised that the communicative methods and timing for exchanging information about the patient was the single most important aspect to classify a partnership as a good one. Success was defined by a quick and simple way of exchanging information about patients and treatment options. Phoning provided a perfect means for these interactions.

*“What I like is that when I send a patient to him, he then talks on the phone with me, tells me what he found, discusses it with me and sends me the results. (…) The cooperation is good. And the treatment of the patients is really good.” (Urologist 14B).*

*“So I make the indication. I discuss it with the patient. I offer the patient to make an appointment with [the collaborating physician]. Sometimes they need some time to think about it. Others say ‘yes, please.‘ So depending on what the patient wants, I call them and if it is urgent I ask to be transferred to the oncologist and discuss the therapy plan immediately with the oncologist.” (Urologist 2B).*

The importance of communication for successful collaboration highlighted joint treatment decisions. Discussions about treatment options were a way for collaborating physicians to recognise another’s competency in their respective field. Such an acknowledgement improved the ways physicians evaluated their collaborations.

*“I take the CT results to discuss with the oncologist. Sometimes we do it during the tumor board and sometimes before. And then I ask him if he would recommend a change in treatment based on the results. We then go back and forth with our opinions and form a decision. The cooperation works because it is not as if he is taking away patients but that we take care of them together and decide jointly who will administer the treatment.” (Urologist 21B).*

## Discussion

In Germany, the establishment of new forms of reimbursement systems was underway to support interdisciplinary cooperation in community settings [21–23]. Their intention was to facilitate and encourage the development of collaborations among physicians. These developments however, were accompanied by tensions between urologists and oncologists [24–26, 28, 32]. While the qualitative interviews showed the development of formal collaborations, the quantitative and qualitative data combined showed it would be misleading to suggest that private practice physicians have not collaborated in the care of their tumor patients previously. Indeed, most surveyed physicians thought that collaborations among physicians are important and many worked together with another physician to deliver optimal treatment to their patients. The most important aspects for a successful and satisfactory cooperation were simple communication structures and knowing one’s partner. The possibility to discuss patients and secure appointments in a timely fashion were factors that facilitated collaborations. The difference in collaboration frequency between urologists residing in western Germany compared to eastern Germany may be inherent to the different health care systems in the former German Democratic Republic (GDR, East) and the former Federal Republic of Germany (FRG, West). Health policy in the GDR was focused on establishing clinics in which a variety of medical specialties worked together (polyclinics and ambulatories), whereas the health care system in the FRG strongly favored single-physician practices, and a strict separation between hospital care and out-patient care predominantly delivered by single-physician private practices. These distinctive infrastructures still play a role in health care delivery in the two geographical regions.

Research that has focused on the collaborations among physicians thus far has focused on general practitioners and their interactions with other specialised and organ-specific physicians. These have identified communication of information between office-based physicians as a barrier to collaborations [33]. In another study communication and accessibility of a physician was identified as influencing physicians’ preferences to refer patients to other physicians [34]. Time and the perception of the competency of other physicians and their ability to serve the patient well have been found as barriers to refer patients from general practice to other medical disciplines [35]. In our study, satisfactory partnerships were associated with the respect that cooperating physicians felt for their partners as well as the clear distribution of roles and responsibilities. In contrast to Belgium, where roles and responsibilities were clearly defined in laws that have restructured the oncological field, such clarity of role definition has not been a major focus in restructuring the German oncological field in community settings [36, 37].

Physicians in this study considered joint treatment decision-making as collaboration. This is the main characteristic of tumor board conferences pertaining to formal collaboration structures [16, 38]. However the study revealed such communication and joint treatment decisions also took place in more informal settings. Physicians involved colleagues in the care of their tumor patients when they considered additional treatments necessary. While there was no standardised way of how physicians decided when this was necessary, this was also true for the more formal collaborations.

The design of the qualitative study assumed a form of collaboration among physicians that did not exist. Indeed, urologists and oncologists worked in a myriad of relationships with many other physicians. There was no single type of collaboration rather it depended on the patient’s wish as much as on the physician’s assessment. Patients have not been the focus of the study however the patient plays a crucial role in decision-making about with whom care is jointly delivered (or not). Ultimately, it was the patient’s choice to accept a recommended physician or not. As the push to more interdisciplinary team approaches moves forward, it is paramount to study patient perspectives on care delivery.

Besides neglecting the patients’ point of view, another limitation was the response rate for the survey (40%). Responders were likely positively attuned to collaboration among physicians. This could explain the positive attitude regarding collaborations in general and the high prevalence of existing collaborations among the respondents. This may also be true for the responders of the qualitative study section. Participants of the interview study were willing to spend several hours in an interview that discussed physician collaboration. Presumably, they had a personal interest in the topic. They also had to be in an existing collaboration with an urologist/oncologist who was also willing to participate in the interview study. This implies both that they want to cooperate and a greater likelihood that they were engaged in a good collaboration. We therefore do not know what barriers would be described by physicians who assess a less positive view regarding collaborations among physicians. Finally, we did not include physicians working in hospital settings since we aimed to learn particularly about private practice collaborations. However, it would be interesting to compare the views of urologists who work in hospital settings and have been collaborating for some time in cancer centers with private practice colleagues. To develop models of collaborations such views may prove important to identify best practices and identify other barriers that hinder good collaboration.

## Conclusion

In the care of their urological tumor patients physicians collaborated in many ways with other physicians to enhance treatment options for patients. Physicians decided when to introduce a patient both formally or informally to other medical professionals. If collaborations among physicians shall be embedded within a more standardised framework it may be important to formulate more clearly which patients should be treated interdisciplinarily, establish simple and direct communication strategies among cooperation partners, and to ensure joint treatment decisions by in part respecting each other’s competencies.
